# Boosting Recovery: Omega-3 and Whey Protein Enhance Strength and Ease Muscle Soreness in Female Futsal Players

**DOI:** 10.3390/nu16244263

**Published:** 2024-12-11

**Authors:** Mina Ahmadi, Niksa Hoorang, Babak Imanian, Mohammad Hemmatinafar, Rasoul Rezaei, Javad Nemati, Fereshte Eftekhari, Walaa Jumah Alkasasbeh

**Affiliations:** 1 Department of Sport Science, Faculty of Education and Psychology, Shiraz University, Shiraz 84334-71946, Iran; 2 Department of Administration and Curriculum, Program of Sports Management and Training, Faculty of Arts and Educational Sciences, Middle East University, Amman 11831, Jordan

**Keywords:** omega-3 and whey protein supplementation, female futsal players, exercise-induced muscle damage (EIMD), delayed-onset muscle soreness (DOMS), strength and power performance

## Abstract

**Background:** Adequate nutrition is crucial for athletes to enhance performance and recovery. This study investigates the acute effects of omega-3 and whey protein supplementation before and after exercise-induced muscle damage (EIMD) on lower-body strength, explosive power, and delayed-onset muscle soreness (DOMS) in female futsal players. **Method:** A randomized, cross-over, placebo-controlled, double-blind study involved 15 female futsal players (Age: 22.93 ± 0.54 years; Height: 159.60 ± 1.16 cm; Weight: 56.95 ± 1.79 kg). Participants completed three conditions: pre-EIMD (1000 mg fish oil, 30 g whey protein, 2 h before EIMD), post-EIMD (same supplementation, within 2 h after EIMD), and placebo (PLA, 2 g starch). EIMD involved 200 vertical jumps with 15% body-weighted vests. Metrics including Sargent jump height (VJH), thigh swelling (Sw-T), pressure pain threshold (PPT), V-sit and reach flexibility test (VSFT), range of motion (ROM), relative peak torque (RPT), average power (AP), and maximal voluntary isometric contraction (MVIC) were recorded 48 h post-EIMD. DOMS was assessed via a visual analog scale (VAS) multiple times. A one-week washout period was employed. **Results:** Pre-EIMD supplementation significantly increased VJH (*p* = 0.001) compared to PLA and Post-EIMD (*p* = 0.033). MVIC45° improved significantly in Pre-EIMD vs. PLA (*p* = 0.001). Improvements were observed in muscle strength metrics, with significant increases in APflx60°/s (pre-EIMD vs. PLA, *p* = 0.001; pre-EIMD vs. post-EIMD, *p* = 0.008), APext60°/s (Pre-EIMD vs. PLA, *p* = 0.030), and APext180°/s (Post-EIMD vs. PLA, *p* = 0.023). DOMS was lower in both Pre-EIMD and Post-EIMD conditions immediately and at 12 h post-EIMD (*p* = 0.009; *p* = 0.030) than PLA. No significant differences were found in Sw-T, PPT, VSFT, ROM, or APflx180°/s. **Conclusions:** Acute omega-3 and whey protein supplementation, particularly before EIMD, improves strength and power and reduces DOMS in female futsal players. Supplement timing may be critical for optimizing recovery and performance in high-demand sports.

## 1. Introduction

Due to the intense nature of futsal, players must be extremely fit to meet the game’s tactical, technical, and physical demands [1]. In futsal matches, players experience repeated high-intensity activities such as tackling, kicking, fast accelerations, and decelerations, along with frequent collisions [2]. Futsal has been ranked among the top ten sports in injury frequency [3], with lower limbs being the primary site of injuries, often resulting from non-contact mechanisms [2]. Furthermore, eccentric contractions with high force or unaccustomed exercises lead to delayed onset muscle soreness (DOMS), contributing to declines in muscle strength and range of motion (ROM) for both athletes and non-athletes [4]. Repetitive eccentric actions are associated with skeletal muscle and deep fascia strain injuries occurring during matches in both men and women [2,5]. Exercise-induced muscle damage (EIMD) can result from such intense activities [5,6].

EIMD is a temporary condition triggered by unaccustomed and strenuous exercise [7]. The most apparent manifestation of EIMD is increased muscle soreness felt 24–72 h after intense exercise. Other symptoms include functional muscle performance losses and localized inflammation [8,9]. The signs of EIMD include pain, inflammation, loss of muscle power and strength, restricted ROM, DOMS, and impaired recovery [10]. Consequently, there is a growing interest in strategies to expedite recovery from vigorous exercise [11]. Athletes often utilize nutritional supplements to mitigate fatigue and enhance performance [12]. Quick recovery to peak performance is primarily sought by those preparing athletes for competitions with multiple tournaments in a short time span [13]. Thus, methods to reduce muscle damage and inflammation post-EIMD are valuable for improving recovery and maintaining performance [11].

Despite the increasing popularity of dietary supplements among athletes, research on their efficacy in futsal players especially in females is limited. One notable supplement for recovery is omega-3 fatty acids [14]. While omega-3s have primarily been studied for cardiovascular health, emerging evidence suggests they may also affect skeletal muscle [15]. Sources of omega-3 fatty acids include fish such as mackerel, salmon, herring, and plant-based options like flax and hemp seeds [16]. Omega-3 fatty acids, particularly docosahexaenoic acid (DHA) and eicosapentaenoic acid (EPA) serve as components of phospholipids in cell membranes and precursors for anti-inflammatory signaling molecules [17]. These fatty acids induce anti-inflammatory effects by modulating the cyclooxygenase-2 and lipoxygenase-5 pathways, thereby suppressing the production of arachidonic acid derivatives [17]. Additionally, omega-3s help regulate pro-inflammatory cytokines, such as tumor necrosis factor-alpha (TNF-α) and interleukin-6 (IL-6), while reducing reactive oxygen species (ROS) production, which contributes to a diminished inflammatory response [18].The anti-inflammatory properties of long-chain polyunsaturated fatty acids (PUFA *n*-3) further support the use of omega-3 dietary supplementation derived from fish oil [15]. Moreover, PUFA *n*-3′s tendency to bind to skeletal muscle cell membranes aids in maintaining membrane integrity, reinforcing the rationale for fish oil consumption in recovery from strenuous exercise [15]. However, studies on the effect of omega-3 supplementation alone on recovery from exercise have yielded mixed results [19,20].

Emerging research highlights additional contexts for omega-3 supplementation in athletes, particularly females. For instance, a study on young active females showed that omega-3 supplementation reduced prostaglandin E2 levels after maximal aerobic exercise, demonstrating its potential to mitigate exercise-induced inflammation [21]. Despite these benefits, many female athletes report suboptimal omega-3 intake, which may hinder their recovery and increase inflammation risk [22]. Additionally, omega-3 supplementation has been associated with reductions in exercise-induced oxidative stress and inflammation, highlighting its role in supporting recovery from physical stress [23]. Despite these findings, there remains a critical gap in understanding omega-3s specific impacts on female athletes, particularly in high-intensity, team-based sports like futsal. Addressing this gap is essential for optimizing nutrition strategies tailored to female athletes’ unique recovery and performance needs.

In addition, whey protein (WP) is a popular supplement that enhances strength and power. Evidence suggests that WP consumption can effectively support muscle contractile function post intense activity and accelerate recovery after resistance training [24]. While amino acid supplementation is sold for promoting recovery after muscle-damaging exercise, the evidence remains equivocal [25]. Recovery from high-intensity exercise is multifaceted, encompassing refueling, repairing, and regenerating skeletal muscles [26]. Thus, optimizing nutritional strategies for muscle recovery should adopt a multidimensional approach, incorporating the synergistic roles of various nutrients [26]. Notably, proteins that facilitate protein synthesis increase following omega-3 supplementation. Although combining EPA and DHA with membrane phospholipids in a fasting state does not influence protein synthesis, it enhances protein synthesis in hyperaminoacidemic and hyperinsulinemic states by indirectly activating the mTOR pathway, a crucial pathway for protein synthesis [27].

The rationale for combining omega-3 with whey protein is supported by studies indicating that omega-3 enhances muscle anabolic sensitivity to amino acid sources [28]. Moreover, due to its amphiphilic properties, whey protein can create a stable layer around oil droplets at the oil–water interface [29]. In one study, Black et al. added fish oil (1546 mg omega-3; 551 mg EPA and DHA) to a protein-based drink over five weeks, finding it to be an effective strategy for reducing muscle pain and fatigue while maintaining better countermovement jump performance in elite rugby players during pre-season training [30]. Similarly, a systematic review by Anthony et al. concluded that omega-3 supplementation positively affects DOMS and inflammatory markers following intense exercise [31]. Conversely, Butler et al. investigated acute omega-3 supplementation’s effects on perceived muscle soreness and recovery after lower-body resistance training, finding no significant benefits compared to placebo [32].

While evidence supports the independent benefits of omega-3 and whey protein supplementation, studies exploring their combined effects remain limited, especially in female athletes. Existing research suggests a potential synergistic effect, with omega-3 enhancing amino acid sensitivity and whey protein stabilizing omega-3 bioavailability [28,30]. For instance, prior studies have indicated reduced muscle soreness and enhanced muscle function with these supplements [31]. However, inconsistencies in the findings highlight the need for further investigation.

The current study seeks to address these gaps by explicitly exploring the acute effects of omega-3 and whey protein supplementation among female futsal players, an underrepresented group in sports nutrition research. Despite the promising potential of these supplements, existing studies have yet to provide conclusive evidence regarding their efficacy in enhancing recovery outcomes such as muscle strength, power, and DOMS. The limited focus on female futsal athletes and the variability in previous findings underscore a significant gap in the literature. Accordingly, this study investigates the acute effects of omega-3 and whey protein supplementation, administered pre- and post-EIMD, on recovery outcomes, including muscle strength, power, and DOMS, aiming to provide actionable insights for optimizing recovery strategies in high-intensity sports.

## 2. Methodology

### 2.1. Participants

Fifteen female futsal players from the Shiraz Premier Women’s Futsal League volunteered to participate in this study. The anthropometric data of the participants is provided in Table 1. Participants self-reported, through health and exercise history questionnaires, that they possessed a minimum of five years of experience playing futsal, had no history of allergy to omega-3 fatty acids or whey protein, and consistently obtained 7 to 8 h of sleep within 24 h. From the number of volunteers who met all of the above conditions, 15 people were selected completely randomly. During the data collection phase, participants indicated that they did not engage in smoking, alcohol consumption, or the intake of caffeine-containing beverages. Before the implementation of the intervention, the study procedures were thoroughly explained, and written informed consent was obtained from each participant. This research was conducted in accordance with the Declaration of Helsinki and received approval from The Human Research Ethics Committee of Shiraz University (Code: IR.US.REC.1402.071); Approval Date: 18 April 2023. Furthermore, all participants were enrolled in the same training camp and followed identical training regimens under the guidance of their trainers. Participants were also instructed to refrain from strenuous exercise 48 h before and after the intervention sessions.

### 2.2. Sample Size Calculation and Study Design

The sample size was determined utilizing the G*Power (version 3.1.9.7) analysis software, with a Type I error rate set at 5% [33]. Based on the prior study conducted by Boutry-Regard et al. in 2020 [34], it was estimated that a total sample of 15 participants would be necessary to discern statistically significant effects in the trial. Consequently, 15 participants were selected for each condition in the current study.

**Figure 1:**
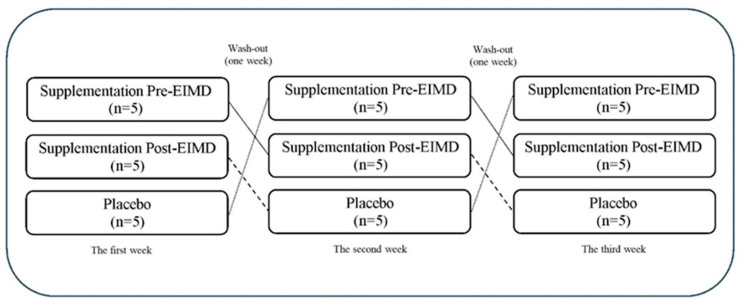
Cross-over and double-blind study design in three conditions.

**Figure 2:**
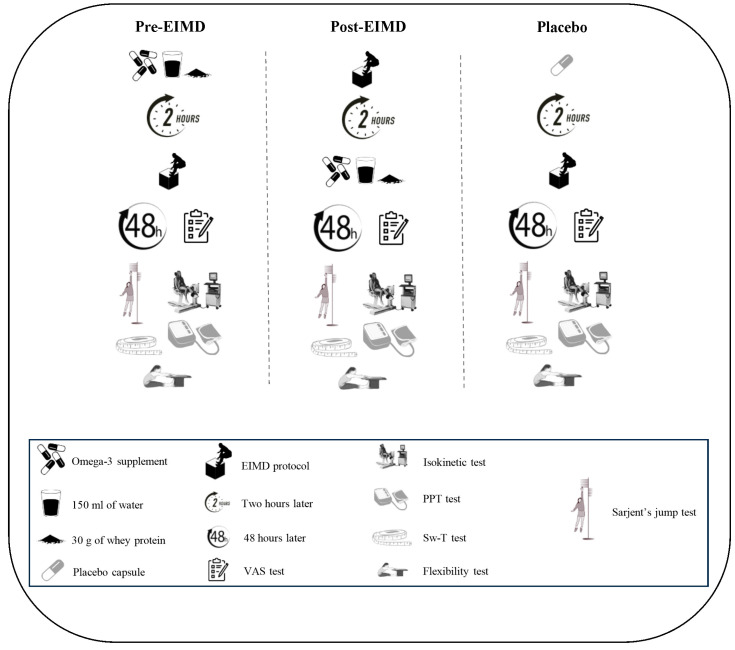
The protocol of taking supplements and performing tests.

This study was conducted in a randomized, cross-over, placebo-controlled, and double-blind design (Figure 1). To ensure consistency, participants attended an initial familiarization session where they were introduced to all testing protocols and procedures, including specific familiarization with the Biodex device and the isokinetic strength test. Each participant underwent three distinct conditions across different sessions: (i) pre-EIMD supplementation (1000 mg fish oil and 30 g whey protein, *n* = 5), administered 2 h before EIMD; (ii) post-EIMD supplementation (1000 mg fish oil and 30 g whey protein, *n* = 5), administered within 2 h following EIMD; and (iii) placebo (PLA, four capsules containing 2 g starch, *n* = 5 (see Figure 2)). In each condition, participants were randomly allocated to one of the intervention arms (5 participants per condition). To clarify, all 15 participants completed each intervention condition in separate experimental sessions, amounting to three unique trials per participant (see Figure 2). The experimental sessions were separated by a one-week washout period to eliminate carryover effects. Delayed onset muscle soreness (DOMS) was assessed using a visual analog scale (VAS) before testing and at 0, 12, 24, and 48 h post-EIMD. Additionally, 48 h post-EIMD, measurements were taken for thigh swelling (Sw-T), pressure pain threshold (PPT), vertical jump height (VJH), V-sit and reach flexibility test (VSFT), range of motion (ROM), and both isokinetic and isometric strength tests. Before each test, participants were asked to abstain from omega-3-rich dietary sources for 24 h to avoid confounding effects. Previous research indicates that the menstrual cycle can significantly influence exercise performance in women [35]. Supplementation and functional tests were conducted during the follicular phase to minimize the effect of hormonal variations occurring during the menstrual cycle on the measured variables. The menstrual cycle phase for each participant was determined using the Menstrual Cycle Questionnaire, and participants with similar cycle timings were selected for the study [36]. Furthermore, all female participants were naturally menstruating and not utilizing any contraceptive methods.

### 2.3. Exercise-Induced Muscle Damage Protocol

Before the EIMD protocol, the participants performed a 10 min warm-up of dynamic movements, slow running, and stretching exercises. Thereafter, participants completed 200 vertical jumps with weighted vests (equivalent to 10% of body weight) to induce muscle damage. For this purpose, each person performed ten sets of 20 maximum jumps (one jump every 4 s). Two minutes of rest (sitting in a chair) were included between sets. To ensure the program’s rigidity, the participants presented and assessed the rate of perceived exertion (RPE) scale immediately after the test. The EIMD protocol was adapted from the previous literature on dietary supplementation [37]. During the study, all participants were members of the same training camp, and their training protocol was the same under the supervision of trainers.

### 2.4. Supplementation Protocol

In the present study, participants ingested four capsules of omega-3 supplement produced in Lugano, Switzerland by the Vivatune company. Each capsule contains 1000 mg of fish oil, which includes 500 mg of EPA and 100 mg of DHA [38]. Additionally, participants consumed 30 g of whey protein supplement powder manufactured by the Karen company in Tehran, Iran, dissolved in 150 mL of water [39]. Participants in the pre-EIMD condition consumed these supplements two hours before the EIMD, while those in the post-EIMD condition ingested them within two hours following the EIMD. The PLA condition involved the consumption of four capsules, each containing 2 g of starch and 150 mL of water, administered two hours before the EIMD.

### 2.5. Examination of Delayed Onset Muscle Soreness by the VAS

The VAS measured the amounts of DOMS. On this scale, a horizontal line of 10 cm is drawn, at the beginning of which the phrase is painless and at the end of which the word is severe pain. VAS is a number that allows a person to express the severity of their pain and is used in experimental and clinical studies. The subjects determined their perception of the severity of delayed onset muscle soreness before the start of the test, immediately, 12, 24, and 48 h after EIMD. By measuring the distance of points marked from the line’s origin with a ruler, the pain score of each person was recorded in millimeters [40]. These time points were chosen based on established research indicating that muscle soreness typically begins within 12 h of high-intensity eccentric exercise, peaks around 24–72 h, and gradually resolves after that [41]. This framework allowed for a comprehensive assessment of the progression and peak of DOMS following the intervention.

### 2.6. Swelling Around the Thigh

The perimeter of the femur was measured three times, ensuring no folds were created in the skin, to assess the degree of swelling surrounding the thigh (Sw-T). A tape measure was utilized, recording measurements to the nearest millimeter. The mean values obtained were documented as the swelling score around the femur. Specific landmarks were marked on the dominant leg to identify the femur’s midpoint accurately. At the same time, the participant was in a standing position, including the greater trochanter of the femur and the tibial prominence [42].

### 2.7. Pressure Pain Threshold

The study determined the pressure pain threshold (PPT) using a blood pressure cuff at the midpoint of the femur (Blood Pressure Cuff—Thigh—Double Tube, MDF Instruments, Rincón, PR, USA). The participants were seated on a chair with their knees bent at a 90-degree angle. A 2.5 cm diameter and 25 cm length plastic tube was placed around the femur midline of the dominant leg. The blood pressure gauge cuff was placed around the participant’s thigh and uniformly inflated. The investigator recorded the pressure level at the onset of pain as the PPT in mmHg [37].

### 2.8. Isokinetic and Isometric Strength Tests

An isokinetic dynamometer (System 4 Pro, Biodex Medical Systems, Inc., Shirley, NY, USA) was used to measure the isokinetic strength of the knee extensor and flexor muscles (concentric phase, at an angular velocity of 60°/s and 180°/s, con/con ratio, dominant leg) with five consecutive repetitions in the direction of extension-flexion and 60 s of rest for recovery between each set. Gravity correction of the torque measurements was accomplished using the Biodex software package (version 4.X). For the tests, participants were stabilized with straps across the chest, above the knee, around the waist, and above the ankle. This arrangement secured the lower leg to the input shaft of the dynamometer. Furthermore, the estimated transverse rotational axis of the knee was visually aligned with the mechanical axis of the dynamometer [43,44,45,46]. Range of motion (ROM), relative peak torque (RPT), and average power (AP) were measured. Maximum voluntary isometric contraction (MVIC) of the dominant leg was measured at 45° and 60° in away (extension) and toward (flexion) action, using the same device. The isometric testing consisted of 5 maximal efforts for 5 s at the knee angles of 45° and 60° [47].

### 2.9. Vertical Jump Height Test

The Sargent Jump test was used to measure vertical jump height (VJH). The participants chalked the end of their fingertips. Then, participants stood the shortest distance from a wall, keeping both feet on the ground, reaching up as high as possible with one hand, and marking the wall with the tips of their fingers (M1). From a static position, the participants jumped as high as possible and marked the wall with the chalk on their fingers (M2). VJH was the distance between M1 and M2. The test was repeated three times with a one-minute passive rest between each attempt, and the immense distance was taken for analysis [48].

### 2.10. V-Sit and Reach Flexibility Test (VSFT)

During the VSFT, participants sat shoeless on the floor with their soles 30 cm apart. A meterstick was placed between the participant’s legs so the 23 cm mark aligned with the participant’s heels [49]. Subjects were later asked to place both hands together and extend forward as far as possible. The best of three attempts was recorded as the final score.

### 2.11. Statistical Analysis

All data were subjected to analysis utilizing both descriptive and inferential statistical methods. The normality of data distribution was assessed through the Shapiro–Wilk test. A repeated measures analysis of variance (ANOVA) was implemented to evaluate the primary effects on functional tests, while a mixed repeated measures ANOVA was applied to ascertain the principal impact on DOMS data. The Bonferroni post hoc test was conducted to identify pairwise differences. Data analysis was performed using SPSS software (version 26, IBM-SPSS Inc., Chicago, IL, USA), with a statistical significance threshold set at *p* ≤ 0.05. GraphPad Prism software (version 9.0, IBM Corporation, San Jose, CA, USA) was also employed for graph design.

## 3. Results

**Figure 3:**
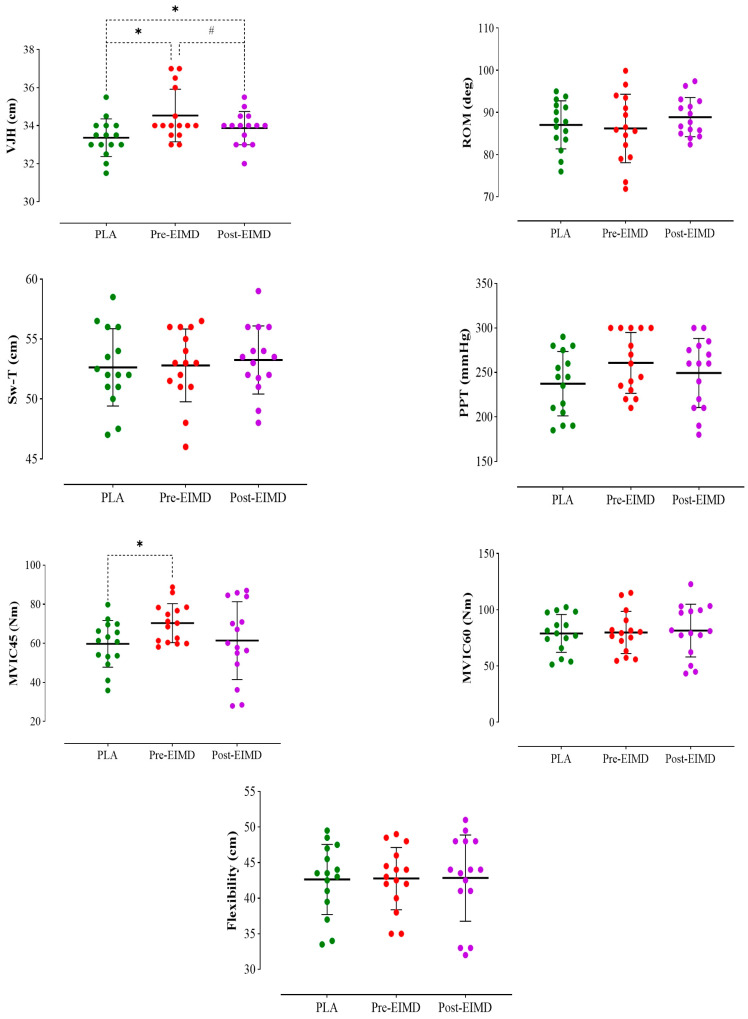
Means and standard deviations of VJH, ROM, MVIC45, MVIC60, Sw-T, PPT, and Flexibility across three conditions. Laboratory experiments were conducted three times, with *n* = 15 participants total and *n* = 5 per group (PLA, pre-EIMD, post-EIMD) in each experiment. *p*-values were calculated using repeated measures analysis of variance (ANOVA) with Bonferroni post hoc tests. *: significant difference compared to PLA. #: significant difference compared to pre-EIMD.

The descriptive stats (incl. mean and SD) are in Table 1. The results showed a significant main effect on VJH (F = 13.632, *p* = 0.001, pη^2^ = 0.493). VJH in pre-EIMD increased vs. PLA (*p* = 0.001) and post-EIMD (*p* = 0.033). No significant difference was found between post-EIMD and PLA (*p* = 0.079) (Figure 3).

The findings indicated a significant main effect on MVIC45° (F = 3.124, *p* = 0.040, pη^2^ = 0.182). Pre-EIMD demonstrated an increase in MVIC45° compared to PLA (*p* = 0.001). However, no significant differences were observed between post-EIMD and PLA (*p* = 1.000) and pre-EIMD (*p* = 0.399) (Figure 3).

**Figure 4:**
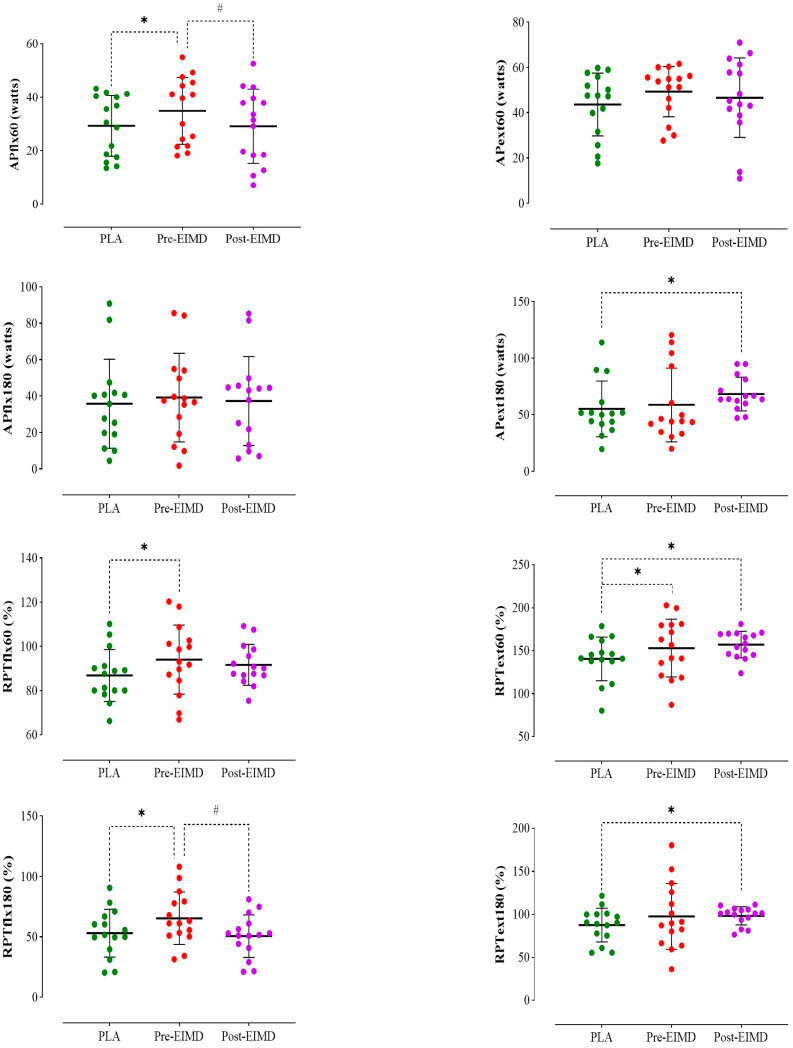
Means and standard deviations of average power during knee flexion and extension at different angular velocities (APflx60°/s, APext60, APflx180, APext180), relative peak torque (RPTflx60, RPText60, RPTflx180, RPText180) across three conditions. Laboratory experiments were conducted three times, with *n* = 15 participants total and *n* = 5 per group (PLA, pre-EIMD, post-EIMD) in each experiment. *p*-values were calculated using repeated measures analysis of variance (ANOVA) with Bonferroni post hoc tests. *: significant difference compared to PLA. #: significant difference compared to pre-EIMD.

The results demonstrate a significant difference among the studied conditions in APflx60°/s, with statistical values indicating F = 13.708, *p* = 0.001, and pη^2^ = 0.495. Specifically, APflx60°/s during the pre-EIMD condition was greater than the PLA condition (*p* = 0.001) and the post-EIMD condition (*p* = 0.008). No significant difference was noted between the post-EIMD and PLA conditions (*p* = 1.000) (Figure 4).

The findings further revealed a noteworthy main effect of supplementation on APext60°/s (F = 2.816, *p* = 0.047, pη^2^ = 0.167). The post hoc tests indicated that APext60°/s in pre-EIMD improved compared to the PLA condition (*p* = 0.030). However, no significant differences were observed between post-EIMD and PLA (*p* = 0.314) or between pre-EIMD and post-EIMD (*p* = 1.000) (Figure 4).

Additionally, the analysis indicated a significant main effect on APext180°/s (F = 3.500, *p* = 0.044, pη^2^ = 0.200), with APext180°/s showing an increase during post-EIMD in comparison to PLA (*p* = 0.023). Nevertheless, no significant differences were found between APext180°/s in post-EIMD and pre-EIMD (*p* = 0.436), or between pre-EIMD and PLA (*p* = 1.000) (Figure 4).

The main effect on RPTflx60°/s was also significant (F = 3.415, *p* = 0.047, pη^2^ = 0.196), with RPTflx60°/s in pre-EIMD exhibiting an increase compared to the PLA condition (*p* = 0.009). No significant differences were observed between post-EIMD and PLA (*p* = 0.148) or between pre-EIMD and post-EIMD (*p* = 1.000) (Figure 4).

A significant main effect of the intervention on RPText60°/s was identified (F = 4.260, *p* = 0.024, pη^2^ = 0.233), with RPText60°/s in post-EIMD (*p* = 0.020) and pre-EIMD (*p* = 0.020) showing improvements relative to PLA. However, there was no significant difference between post-EIMD and pre-EIMD (*p* = 1.000) (Figure 4).

Furthermore, the results indicated a significant main effect of supplementation on RPTflx180°/s (F = 13.378, *p* = 0.001, pη^2^ = 0.489), with RPTflx180°/s in pre-EIMD increased compared to PLA (*p* = 0.002) and post-EIMD (*p* = 0.009). However, no significant difference was found between post-EIMD and PLA (*p* = 0.493) (Figure 4).

The main effect of the intervention on RPText180°/s was shown to be substantial (F = 1.361, *p* = 0.049, pη^2^ = 0.089), with RPText180°/s in post-EIMD being higher than that of PLA (*p* = 0.024). Nevertheless, no significant differences were observed between post-EIMD and pre-EIMD (*p* = 1.000) and between pre-EIMD and PLA (*p* = 0.609) (Figure 4).

The analysis exhibited no significant differences across the three studied conditions in Sw-T (F = 1.286, *p* = 0.292, pη^2^ = 0.084), PPT (F = 3.053, *p* = 0.063, pη^2^ = 0.179), VSFT (F = 0.094, *p* = 0.806, pη^2^ = 0.007), ROM (F = 1.159, *p* = 0.328, pη^2^ = 0.076), APflx180°/s (F = 0.967, *p* = 0.368, pη^2^ = 0.065), or MVIC60° (F = 0.203, *p* = 0.740, pη^2^ = 0.014) (see Figure 3 and Figure 4). All relevant data are provided in Table 2 and Table 3.

The means and standard deviations of the DOMS reports are outlined in Table 4. The results further indicated a significant main effect on DOMS immediately following EIMD (F = 6.692, *p* = 0.005, pη^2^ = 0.320). Specifically, DOMS immediately post-EIMD in the pre-EIMD condition was lower than that in the PLA condition (*p* = 0.018); however, no significant differences were identified between post-EIMD and PLA (*p* = 0.200) or between pre-EIMD and post-EIMD (*p* = 0.224).

**Figure 5:**
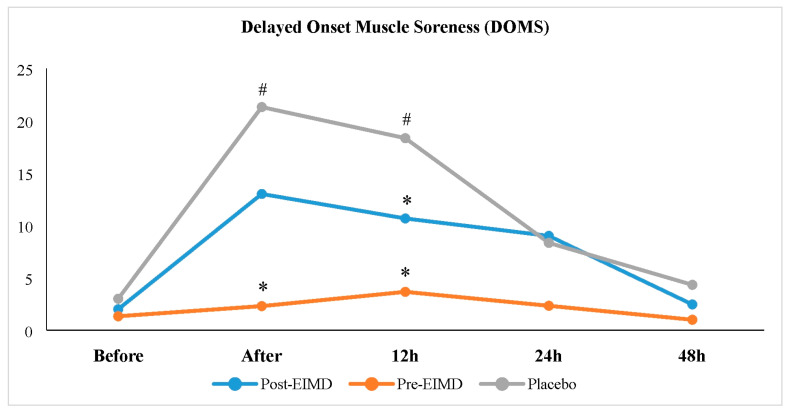
Changes in average delayed onset muscle soreness (DOMS) at different time points (immediate, 12 h, 24 h, and 48 h post-EIMD) across three conditions. *p*-values were calculated using mixed repeated measures ANOVA with Bonferroni post hoc tests. *: significant difference compared to PLA. #: significant difference compared to DOMS before EIMD.

The analysis indicated a significant difference among the studied conditions in DOMS recorded twelve hours following EIMD (F = 8.856, *p* = 0.001, pη^2^ = 0.387), with DOMS twelve hours after EIMD being reduced in post-EIMD (*p* = 0.030) and pre-EIMD (*p* = 0.009) relative to PLA. Nonetheless, no significant difference was observed between post-EIMD and pre-EIMD conditions (*p* = 0.219). However, the results showed no significant differences in DOMS before EIMD (F = 0.780, *p* = 0.468, pη^2^ = 0.053) or at 24 h (F = 2.485, *p* = 0.131, pη^2^ = 0.151) and 48 h (F = 1.299, *p* = 0.277, pη^2^ = 0.085) after EIMD among the studied conditions (Table 5 and Figure 5).

The findings of the Bonferroni test revealed that, within the PLA condition, DOMS immediately (*p* = 0.049) and twelve hours (*p* = 0.050) post-EIMD were significantly higher compared to DOMS before EIMD. However, no significant differences were noted between DOMS at 24 h (*p* = 1.000) and 48 h (*p* = 1.000) relative to DOMS before EIMD (Table 5 and Figure 5).

In the post-EIMD condition, no significant differences were observed in DOMS immediately (*p* = 0.733), twelve hours (*p* = 0.294), twenty-four hours (*p* = 1.000), or forty-eight hours (*p* = 1.000) after EIMD compared to DOMS before EIMD (see Table 5 and Figure 5).

The results indicated that in the pre-EIMD condition, there were no significant differences in DOMS immediately (*p* = 1.000), twelve hours (*p* = 1.000), twenty-four hours (*p* = 1.000), or forty-eight hours (*p* = 1.000) post-EIMD when compared to DOMS before EIMD (see Table 5 and Figure 5).

## 4. Discussion

This study evaluated the impact of an acute administration of omega-3 fatty acids and whey protein on strength, muscle power, and the recovery response following delayed onset muscle contusion in female futsal players. We hypothesized that omega-3 supplementation could serve as an effective strategy for mitigating muscle inflammation and promoting functional recovery [50]. Moreover, omega-3 supplementation has demonstrated efficacy in recovery and inflammation reduction, is widely accessible, cost-effective, and may positively influence muscle protein synthesis [27,28]. Furthermore, the significance of protein intake among athletes must be noticed, as it is widely accepted that an increase in dietary protein for football and futsal players enhances strength accelerates recovery, and consequently improves both endurance and non-endurance performance [51]. Thus, combining omega-3 and whey protein supplements may provide substantial benefits regarding DOMS, muscle power, and overall strength. While the results indicate potential benefits of pre-EIMD supplementation, the findings should be viewed as preliminary given the limited sample size and specific context of this study.

The present study’s findings indicated a significant increase in vertical jump height (VJH) in the pre-EIMD condition compared to the placebo group (PLA). Additionally, an improvement was observed in the pre-EIMD condition relative to the post-EIMD condition. These results align with several previous studies. For instance, in a supplement group, countermovement jump (CMJ) height increased by 2.2%, while a decline of 1.1% in CMJ performance was noted in the PLA group [52]. Moreover, research conducted by Black et al. on elite rugby players demonstrated that supplementation with omega-3 fatty acids (1.1 g∙d^−1^ EPA + DHA) over five weeks led to a 4.6% enhancement in CMJ performance, contrasted with a 3.4% decrease in the PLA group [30]. Conversely, some previous studies reported findings inconsistent with the current study’s results. Various studies indicated reductions in peak power generation, jump performance, and time trial outcomes [53]. Omega-3 supplementation, particularly with a protein-based drink, may influence sleep, stress, and mood. These findings suggest that administering a protein-based drink containing omega-3 polyunsaturated fatty acids (PUFA) twice daily may alleviate lower body muscle soreness and fatigue, thus better preserving neuromuscular performance [30]. Furthermore, omega-3 fatty acids may exert a beneficial effect on muscle protein synthesis [27]. The observed improvement in VJH in the pre-EIMD condition may be influenced by factors such as the acute supplementation protocol, participant variability, external conditions, and the small sample size. Despite these limitations, the findings hold significant implications for futsal players, highlighting the potential of omega-3 and whey protein supplementation to reduce muscle soreness and fatigue while supporting performance during short recovery periods between training or matches. Future studies with larger samples and extended interventions must confirm and further investigate these effects.

The present study’s findings indicate that the acute consumption of omega-3 and whey protein supplements, administered before and after a traumatic exercise session, does not significantly affect Sw-T in female futsal players. However, the results suggest that following EIMD, the circumference of the thigh is higher than in the pre-EIMD intervention condition. Although this difference does not reach statistical significance, it implies that the inflammatory response in the thigh muscles of the participants during the pre-EIMD condition was less severe, as evidenced by a 5.3% change in thigh area in the post-EIMD condition compared to 1.6% in the placebo group, and 1.5% in the pre-EIMD condition. Supporting the current study, Ayubi et al., 2022 demonstrated that omega-3 supplementation significantly diminishes serum levels of TNF-α, indicating a reduction in inflammation [54]. Furthermore, Nieman et al., 2020 reported that whey protein supplementation significantly lessened post-exercise blood levels of muscle damage biomarkers relative to water alone [55]. In this study, omega-3 supplementation effectively mitigated muscle inflammation by down-regulating pro-inflammatory cytokines such as TNF-α and IL-6, thereby reducing the production of arachidonic acid (AA) and reactive oxygen species (ROS). Increasing evidence supports the idea that omega-3 supplementation can attenuate the production of pro-inflammatory cytokines and ROS, suggesting a direct correlation between recovery from intense exercise and the associated inflammatory markers [11]. Moreover, whey protein may reduce inflammation by converting the amino acid cysteine into glutathione, a potent intracellular antioxidant, and stimulating muscle protein synthesis (MPS) [24]. Although Sw-T during the pre-EIMD condition was lower than that observed post-EIMD in female futsal players, this difference did not achieve statistical significance. The lack of significance may be attributed to the supplements’ dosage or the relatively small sample size compared to other studies.

Muscle stiffness and swelling resulting from EIMD have been shown to impede ROM following physical exertion [56]. The present study’s findings indicate no significant changes in knee ROM under any conditions assessed, including pre-EIMD, post-EIMD, and placebo (PLA) following muscle-damaging exercise. However, a slight difference was observed in post-EIMD compared to both pre-EIMD and PLA. These results align with previous research; for instance, a study demonstrated that an 8-week supplementation of omega-3 fatty acids (58) inhibited declines in muscle strength, reduced ROM, and alleviated muscle soreness [57]. Thus, the neurological effects associated with omega-3 may contribute to the attenuation of muscle damage following EIMD [57]. The limited sample size and financial constraints impacting access to neurophysiological studies may account for the discrepancies observed in the current findings. Furthermore, the results of the present study revealed that the MVIC45° in pre-EIMD significantly increased compared to PLA. However, several studies have reported no significant differences in MVIC between groups, nor was any interaction effect observed, suggesting that the levels of muscle damage were unaffected by omega-3 supplementation [11,58]. Our findings regarding the increased MVIC45° following supplementation align with recent research highlighting the role of omega-3 fatty acids in reducing inflammation and facilitating recovery. For example, Ayubi et al., 2022 demonstrated that omega-3 intake reduces inflammatory cytokine responses [54], enabling athletes to regain strength and muscle function more rapidly than those not receiving supplementation. This enhancement in recovery is attributed to the influence of omega-3 fatty acids on inflammatory mediators such as TNF-α and IL-6, which are known to compromise muscle function when present at elevated levels following intense exertion.

The results of the present study indicate a significant increase in APflx60°/s during the pre-EIMD condition compared to both the PLA and post-EIMD conditions. Moreover, a notable rise in APext60°/s was observed in the pre-EIMD condition relative to the PLA group. Additionally, APext180°/s demonstrated a significant improvement in the post-EIMD condition compared to PLA. However, no significant differences were observed between the conditions in APflx180°/s. In agreement with the current study’s findings, Jakeman et al. reported a significant time main effect for knee extensor strength at both 60° and 180° [8]. This suggests that the effects of omega-3 supplementation on muscle soreness may be attributed to the down-regulation of pro-inflammatory cytokines, such as TNF-α and IL-6, which in turn reduces the production of arachidonic acid (AA) and reactive oxygen species (ROS), thereby diminishing the inflammatory response [18]. Furthermore, protein ingestion has been shown to stimulate protein synthesis through the mechanistic target of the rapamycin (mTOR) pathway, consequently enhancing muscle mass and strength [59]. The results of the present study also revealed that RPTflx60°/s exhibited a significant increase in the pre-EIMD condition compared to the PLA group. Additionally, RPTflx180°/s was significantly higher in the pre-EIMD condition than in both the PLA and Post-EIMD conditions. RPText60°/s demonstrated improvements in the pre-EIMD and Post-EIMD conditions compared to the PLA group. Conversely, RPText180°/s significantly increased the post-EIMD condition relative to PLA. Therefore, the co-ingestion of omega-3 and whey protein may enhance muscle strength, as mentioned earlier.

The current study’s findings indicate that the intervention did not significantly impact VSFT and PPT in female futsal players. Furthermore, the research demonstrated that the acute intake of omega-3 and whey protein supplements before and after EIMD significantly influenced the occurrence of DOMS. This effect was notably evident in pre-EIMD and post-EIMD conditions compared to the PLA group, particularly in immediately and 12 h post-EIMD. As illustrated in Figure 4, the levels of DOMS in the pre-EIMD and post-EIMD periods exhibited a more pronounced decrease over the 48 h recovery window. Even within the PLA conditions, a significant variance was observed in the escalation of DOMS immediately post-exercise and 12 h after the EIMD protocol, in contrast to the baseline measurements taken before the EIMD. These findings are supported by a study that examined the effects of whey protein supplementation on muscle damage and the severity of delayed-onset muscle soreness following acute maximal weightlifting exercises. Although the administration of a protein supplement at a dose of 1.5 g/kg did not significantly reduce creatine kinase (CK) and myoglobin (Mb) levels after weight-lifting, the research indicated that DOMS was reduced substantially [60]. Similarly, another investigation concluded that omega-3 supplementation positively affected DOMS and inflammatory markers resulting from atypical exercise [61]. Conversely, some studies yielded results that did not agree with these findings. For instance, Lass et al. examined the effects of omega-3 supplementation administered after a resistance training session in women maintaining a balanced diet, finding no significant interactions across measured outcomes. Participants reported heightened muscle soreness and diminished muscle power capacity within three days post-exercise, with no differences observed between the placebo and omega-3 groups [62]. Additionally, Butler et al., 2019 [32] assessed the impact of acute omega-3 supplementation on perceived muscle soreness, activity levels, and recovery following a lower body resistance training protocol among university-aged males. Their results indicated that omega-3 supplementation did not significantly enhance the evaluated variables compared to placebo [32]. In summary, the data suggest that acute supplementation with omega-3 and whey protein can be advantageous for mitigating DOMS among female futsal players following intense physical activity, particularly during the immediate hours after exercise. While the intervention did not significantly influence flexibility or PPT, the observed reduction in DOMS may facilitate faster recovery and enhance readiness for subsequent training or competition. These results are consistent with the literature, highlighting the anti-inflammatory and muscle recovery properties of omega-3 and protein supplements. Nevertheless, inconsistent outcomes across similar studies indicate that variables such as supplement dosage, timing, exercise protocol, and participant characteristics may significantly affect efficacy, pointing to the necessity for individualized approaches in optimizing recovery strategies. Also, it is essential to note that the 15-participant sample size limits the power to detect more minor or nuanced effects. Thus, these findings may not fully represent the responses in broader athletic populations.

As previously articulated, following a rigorous exercise protocol, an elevation in creatine kinase (CK) levels is observed beginning approximately 24 h post-exercise, peaking between 48 and 72 h. This elevation serves as an established biomarker for muscle damage [63]. Armstrong and Smith have posited that injury to connective or contractile tissue triggers an inflammatory response. During this response, monocytes migrate to the injured site, differentiating into macrophages, and subsequently secrete significant quantities of pro-inflammatory prostaglandins [64,65]. The elevation of prostaglandins, coupled with increases in histamines, kinins, and peripheral edema, activates pain receptors (specifically, group III and IV afferents), resulting in the sensation of DOMS [64,65]. Research has demonstrated that omega-3 polyunsaturated fatty acids, namely DHA and EPA, exert anti-inflammatory effects by modulating the cyclooxygenase 2 (COX-2) and lipoxygenase five pathways. Omega-3 fatty acids are critical components of phospholipids within cell membranes and serve as precursors for producing anti-inflammatory signaling molecules. The most recognized omega-3 fatty acids include alpha-linolenic acid (ALA), EPA, and DHA [17]. A significant association between omega-3 fatty acids and muscle inflammation is established through the down-regulation of pro-inflammatory cytokines such as tumor TNF-α) and IL-6, as well as the reduction in ROS production. This cascade of events contributes to a diminished inflammatory response [18]. Furthermore, omega-3 fatty acids enhance muscle anabolic sensitivity to amino acid sources [28]. The amphiphilic properties of whey protein allow it to adsorb at the oil–water interface, forming a high-resistance surface layer around oil droplets [29]. In the present study, the principal reason for the observed reduction in DOMS under pre-EIMD and post-EIMD conditions, in contrast to the PLA condition, can be attributed to the factors above. Notably, the rate of DOMS reduction in pre-EIMD conditions is more pronounced than in post-EIMD and PLA conditions. Consequently, omega-3 and whey protein supplementation before participating in an EIMD protocol among female futsal players dramatically alleviates the sensation of DOMS. Moreover, it was anticipated that the intake of omega-3 and whey protein supplements before and after strenuous training would positively impact the VSFT and pain PPT in this population by mitigating inflammation and delayed muscle soreness. However, this anticipated effect was not observed, which may be attributed to the limited sample size and the influence of uncontrollable variables.

This study has several noteworthy limitations that should be considered when interpreting the findings. First, the sample size was limited to 15 female futsal players, which, although determined based on G*Power calculations, reduces the statistical power to detect more minor or nuanced effects. This number also falls short of representing the total composition of a futsal team, which typically includes 14–16 players, further constraining the generalizability of the results to larger athletic populations. Additionally, the small sample size was due to the need for consistent training under a single coach, which restricted recruiting more participants. Second, due to financial constraints and logistical challenges, we could not include analyses of critical inflammatory and muscle damage biomarkers, such as CK, IL-6, and TNF-α. These biomarkers could have provided more robust mechanistic insights into the physiological pathways underpinning the observed effects. The high costs associated with importing specialized kits for biomarker analyses exceeded our limited research budget. Finally, this study’s short duration of supplementation limits insights into the potential long-term effects of omega-3 and whey protein supplementation on recovery and performance. Future research should include extended intervention periods and more diverse samples to understand these effects comprehensively. Despite these limitations, this study offers valuable preliminary insights into the potential benefits of omega-3 and whey protein supplementation in female futsal players. We strongly recommend future studies, possibly with international collaborations or funding support, to address these limitations and further explore the mechanisms underlying the observed effects.

## 5. Conclusions

This study demonstrated that acute omega-3 and whey protein supplementation effectively reduced DOMS and enhanced recovery metrics in female futsal players following EIMD, with notable reductions immediately and 12 h post-exercise. Pre-EIMD supplementation showed more pronounced benefits in reducing DOMS and improving strength and power metrics than post-EIMD supplementation, highlighting the critical role of supplement timing in maximizing recovery benefits. While no significant improvements were observed in certain flexibility and pain thresholds (e.g., VSFT or PPT), these supplements enhanced overall recovery and potentially improved readiness for subsequent activity. These findings underscore the potential of omega-3 and whey protein supplementation to support recovery and performance readiness in high-intensity sports, particularly for female athletes, addressing a significant gap in sports nutrition research. They also offer practical applications for optimizing recovery strategies in sports with frequent competition and training sessions. Future research with larger, more diverse samples and extended supplementation durations is encouraged to confirm and generalize these outcomes and explore the long-term effects of such interventions.

## Figures and Tables

**Table 1:** The anthropometric data of participants.

Characteristic	Mean ± SD (*n* = 15)
**Age (years)**	22.93 ± 0.54
**Height (cm)**	159.60 ± 1.16
**Weight (kg)**	56.95 ± 1.79
**BMI (kg/m^2^)**	22.21 ± 0.52

**Table 2:** Mean and standard deviation (Mean ± SD) of measured variables (*n* = 15).

Variables	PLA	Pre-EIMD	Post-EIMD
**VJH (cm)**	33.36 ± 0.99	34.53 ± 1.38	33.86 ± 0.87
**Sw-T (cm)**	52.63 ± 3.23	52.80 ± 3.03	53.25 ± 2.84
**PPT (mmHg)**	237.33 ± 36.14	260.66 ± 34.16	249.33 ± 38.86
**VSFT (cm)**	42.63 ± 4.91	42.76 ± 4.37	42.83 ± 6.05
**APflx60°/s (watts)**	29.34 ± 11.35	34.92 ± 12.47	29.15 ± 13.87
**APext60°/s (watts)**	43.60 ± 13.86	49.30 ± 11.08	46.59 ± 17.56
**APflx180°/s (watts)**	35.80 ± 24.40	39.20 ± 24.30	37.30 ± 24.35
**APext180°/s (watts)**	55.18 ± 24.55	58.76 ± 32.50	68.32 ± 14.89
**RPTflx60°/s (%)**	86.88 ± 11.73	94.02 ± 15.59	91.68 ± 9.24
**RPText60°/s (%)**	140.54 ± 25.44	153.03 ± 33.68	157.18 ± 15.43
**RPTflx180°/s (%)**	53.08 ± 19.80	65.39 ± 21.76	50.60 ± 17.65
**RPText180°/s (%)**	87.55 ± 19.56	97.62 ± 38.21	98.26 ± 10.60
**MVIC45° (Nm)**	59.73 ± 11.94	70.34 ± 9.97	61.39 ± 19.93
**MVIC60° (Nm)**	78.94 ± 16.79	79.86 ± 18.74	81.50 ± 23.45
**ROM (deg)**	87.06 ± 5.69	86.20 ± 8.13	88.89 ± 4.61

Pre-EIMD: before exercise-induced muscle damage; post-EIMD: after exercise-induced muscle damage; PLA: placebo; VJH: Sargent’s jump height; Sw-T: swelling around the thigh; PPT: pressure pain threshold; AP: average power; RPT: relative peak torque; flx: flexion; ext: extension; MVIC: maximal isometric voluntary contraction; ROM: range of motion; cm: centimeter; Nm: Newton meters; deg: degree.

**Table 3:** Pairwise comparisons in the three conditions (*n* = 15).

		Pre-EIMD		Post-EIMD	
		PLA	Post-EIMD	PLA	Pre-EIMD
**VJH (cm)**	**MD**	1.16	0.66	0.50	−0.66
	**Sig**	0.001	0.033	0.079	0.033
	**95% CI**	0.50–1.82	0.04–1.28	−0.04–1.04	−1.28–−0.04
**Sw-T (cm)**	**MD**	0.16	−0.45	0.61	0.45
	**Sig**	1.000	1.000	0.271	1.000
	**95% CI**	−0.90–1.24	−1.67–0.77	−0.30–1.53	−0.77–1.67
**PPT (mmHg)**	**MD**	23.33	11.33	12.00	−11.33
	**Sig**	0.061	0.895	0.688	0.895
	**95% CI**	1.19–45.47	−17.18–39.85	−13.94–37.94	−39.85–17.18
**VSFT (cm)**	**MD**	0.13	−0.06	0.20	0.06
	**Sig**	1.000	1.000	1.000	1.000
	**95% CI**	−0.59–0.86	−1.75–1.62	−1.02–1.42	−1.62–1.75
**APflx60°/s (watts)**	**MD**	5.58	5.77	−0.18	−5.77
	**Sig**	0.000	0.008	1.000	0.008
	**95% CI**	2.61–8.56	1.45–10.09	−2.87–2.50	−10.09–−1.45
**APext60°/s (watts)**	**MD**	5.70	2.71	2.99	−2.71
	**Sig**	0.030	1.000	0.314	1.000
	**95% CI**	0.50–10.91	−6.18–11.61	−1.69–7.68	−11.61–6.18
**APflx180°/s (watts)**	**MD**	3.40	1.89	1.50	−1.89
	**Sig**	0.287	1.000	1.000	1.000
	**95% CI**	−1.77–8.57	−6.65–10.43	−4.26–7.27	−10.43–6.65
**APext180°/s (watts)**	**MD**	3.58	−9.55	13.13	9.55
	**Sig**	1.000	0.436	0.023	0.436
	**95% CI**	−9.39–16.55	−26.38–7.27	1.63–24.63	−7.27–26.38
**RPTflx60°/s (%)**	**MD**	7.14	2.34	4.80	−2.34
	**Sig**	0.009	1.000	0.148	1.000
	**95% CI**	1.70–12.57	−7.93–12.61	−1.26–10.86	−12.61–7.93
**RPText60°/s (%)**	**MD**	12.49	−4.14	16.64	4.14
	**Sig**	0.020	1.000	0.020	1.000
	**95% CI**	1.79–23.19	−25.69–17.40	2.44–30.83	−17.40–25.69
**RPTflx180°/s (%)**	**MD**	12.31	14.78	−2.47	−14.78
	**Sig**	0.002	0.009	0.493	0.009
	**95% CI**	4.50–20.12	3.57–26.00	−7.05–2.10	−26.00–−3.57
**RPText180°/s (%)**	**MD**	10.07	−0.64	10.71	0.64
	**Sig**	0.609	1.000	0.024	1.000
	**95% CI**	−10.42–30.57	−26.45–25.17	1.26–20.16	−25.17–26.45
**MVIC45° (Nm)**	**MD**	10.60	8.94	1.66	−8.94
	**Sig**	0.001	0.399	1.000	0.399
	**95% CI**	4.66–16.55	−6.29–24.18	−12.27–15.59	−24.18–6.29
**MVIC60° (Nm)**	**MD**	0.88	−1.67	2.56	1.67
	**Sig**	1.000	1.000	1.000	1.000
	**95% CI**	−6.14–7.91	−15.28–11.94	−9.00–14.12	−11.94–15.28
**ROM (deg)**	**MD**	−0.85	−2.68	1.83	2.68
	**Sig**	1.000	0.545	0.666	0.545
	**95% CI**	−6.32–4.16	−7.88–2.50	−2.06–5.73	−2.50–7.88

Pre-EIMD: before exercise-induced muscle damage; post-EIMD: after exercise-induced muscle damage; PLA: placebo; MD: mean difference; CI: confidence interval; VJH: Sargent’s jump height; Sw-T: swelling around the thigh; PPT: pressure pain threshold; AP: average power; RPT: relative peak torque; flx: flexion; ext: extension; MVIC: maximal voluntary contraction; ROM: range of motion; cm: centimeter; Nm: Newton meters; deg: degree.

**Table 4:** Mean and standard deviation (Mean ± SD) of the DOMS in each condition (*n* = 15).

Variables	PLA	Pre-EIMD	Post-EIMD
**DOMS before (cm)**	3.00 ± 4.14	1.33 ± 2.96	2.00 ± 3.16
**DOMS after (cm)**	21.33 ± 21.66	2.33 ± 4.57	13.00 ± 20.07
**DOMS 12 h (cm)**	18.33 ± 16.10	3.66 ± 5.81	10.66 ± 14.37
**DOMS 24 h (cm)**	8.33 ± 13.97	2.33 ± 6.77	9.00 ± 16.16
**DOMS 48 h (cm)**	4.33 ± 10.49	1.00 ± 2.80	2.46 ± 7.73

Pre-EIMD: before exercise-induced muscle damage; Post-EIMD: after exercise-induced muscle damage; PLA: placebo; DOMS: delayed onset muscle soreness; before: before EIMD; after: immediately after EIMD; 12 h: 12 h after EIMD; 24 h: 24 h after EIMD; 48 h: 48 h after EIMD.

**Table 5:** Pairwise comparisons of the DOMS in the three conditions (*n* = 15).

		Pre-EIMD		Post-EIMD	
		PLA	Post-EIMD	PLA	Pre-EIMD
**DOMS before (cm)**	**MD**	−0.86	−6.33	5.46	6.33
	**Sig**	1.000	1.000	0.618	1.000
	**95% CI**	−18.09–16.36	−25.84–13.17	−5.73–16.66	−13.17–25.84
**DOMS after (cm)**	**MD**	−19.00	−10.66	−8.33	10.66
	**Sig**	0.018	0.224	0.200	0.224
	**95% CI**	−34.92–3.07	−25.72–4.38	−19.71–3.04	−4.38–25.72
**DOMS 12 h (cm)**	**MD**	−14.66	−7.00	−7.66	7.00
	**Sig**	0.009	0.219	0.030	0.219
	**95% CI**	−25.79–−3.53	−16.81–2.81	−14.66–−0.66	−2.81–16.81
**DOMS 24 h (cm)**	**MD**	−6.00	−6.66	0.66	6.66
	**Sig**	0.351	0.401	1.000	0.401
	**95% CI**	−15.76–3.76	−18.04–4.71	−3.28–4.61	−4.71–18.04
**DOMS 48 h (cm)**	**MD**	−3.33	−1.46	−1.86	1.46
	**Sig**	0.735	1.000	0.233	1.000
	**95% CI**	−10.79–4.12	−7.16–4.22	−4.53–0.79	−4.22–7.16

Pre-EIMD: before exercise-induced muscle damage; post-EIMD: after exercise-induced muscle damage; PLA: placebo; MD: mean difference; CI: confidence interval; DOMS: delayed onset muscle soreness; before: before EIMD; after: immediately after EIMD; 12 h: 12 h after EIMD; 24 h: 24 h after EIMD; 48 h: 48 h after EIMD.

## Data Availability

The datasets used and/or analyzed during the current study are available from the corresponding author upon reasonable request. The data are not publicly available due to technical/time limitations.
